# Morphological and Ultrastructural Characterization of the Venom Apparatus of the Predatory Stink Bug, *Arma custos*

**DOI:** 10.3390/insects17030340

**Published:** 2026-03-20

**Authors:** Yuqin Wang, Ping Gao, Chaoyan Wu, Wenxiu Wang, Jiaying Zhu

**Affiliations:** 1 Key Laboratory of Forest Disaster Warning and Control of Yunnan Province, Southwest Forestry University, Kunming 650224, China; wangyuqin2022@swfu.edu.cn (Y.W.); gaoping65432@163.com (P.G.); wcy1316033@swfu.edu.cn (C.W.); wangwenxiu@swfu.edu.cn (W.W.); 2 Key Laboratory for Forest Resources Conservation and Utilization in the Southwest Mountains of China, Ministry of Education, Southwest Forestry University, Kunming 650224, China

**Keywords:** venom apparatus, morphology, secretory cell, ultrastructure, predatory bug

## Abstract

The predatory bug *Arma custos* (Hemiptera: Pentatomidae) is a natural enemy insect capable of preying on over 40 types of agricultural and forestry pests. Here, we describe the characteristics of the morphology and ultrastructure of its venom apparatus visualized using light and electron microscopy. Light microscopy revealed that the venom apparatus of *A. custos* consists of a pair of main gland and tubular accessory gland. The main gland consist of two lobes, the anterior main gland (AMG) and posterior main gland (PMG). Between the two lobes of the main gland, there is a strong constriction, characterizing a hilum (Hi) where two separate ducts, the venom duct of the main gland (VD) and the duct connecting the accessory gland to the main gland (AMD), are inserted. The VD extends toward the head and connects to the venom pump (VP), while the AMD extends toward the thorax and connects to the accessory gland (AG). Ultrastructural examination of the venom glands reveals that the AMG and PMG consist of a layer of cubic or spherical glandular cells forming a large circular lumen, while the AG exhibits two narrow lumens. The secretory cytoplasm of AMG, PMG, and AG contains a well-developed rough endoplasmic reticulum, along with mitochondria, nuclei, secretory vesicles, autophagosomes, and secretory granules. However, significant differences exist in the ultrastructural characteristics among the three glands. Unlike glandular secretory cells in the venom glands, the ultrastructure of VD, and AMD reveals only well-developed nuclei, mitochondria, and elaborate plasma membrane folds. These results indicate that venom proteins are synthesized and stored by the AMG, PMG, and AG, while the VD and AMD ducts are responsible for transporting the venom.

## Simple Summary

In this work, we characterized the morphology and ultrastructure of the venom apparatus in *Arma custos* using light and electron microscopy. Our study found that the venom apparatus of *A. custos* consists of glands and ducts. The glandular cells in the AMG, PMG, and AG display typical secretory cell architecture and function to secrete venom, whereas the duct cells in the VD and AMD lack secretory structures and are primarily involved in venom transport. However, the differences in organelle type, shape, size, and electron density among the three glandular cells are the real reason why the venom composition and function of the AMG, PMG, and AG differ.

## 1. Introduction

Heteropterans with predatory and bloodsucking habits are recognized as venomous animals, possessing a unique venom apparatus called salivary glands, which differs from that composed of a venom gland and a reservoir found in Hymenoptera [1,2,3,4]. The venom apparatus of heteropterans consists of paired main glands (MGs) and accessory glands (AGs) [5,6]. The main glands are bilobed, the anterior main gland (AMG) and posterior main gland (PMG), with a noticeable constriction between the two lobes, forming a hilum (Hi), where two separate ducts, named the venom duct of the main gland (VD) and the duct connecting the accessory gland to the main gland (AMD), are inserted [5,6,7]. The AMD duct extends towards the thoracic cavity, expanding to form accessory gland [8,9,10,11,12,13,14]. Extensive reviews are available on the morphology, histology, and ultrastructure of the venom apparatus of hemipteran insects [7,8,9,10,11,12,13,14]. Morphological analysis indicates that MGs exhibit single-lobed, bilobed, trilobed, and multilobed morphology, while the AGs display diverse morphologies such as tubular, vesicular, and lamellar structures [7,8,10,14]. Histological and ultrastructural characterization of the AMG, PMG, and AG structures indicate that they are formed by a single secretory epithelium covering a lumen where venom is stored [10,11,12,14]. However, these studies have primarily focused on the AMG, PMG, and AG, while the ductal structures responsible for venom transport remain to be well elucidated.

Heteropteran venom is a cocktail of proteinaceous and chemical compounds. It serves as a chemical weapon for predation, defense, and competition deterrence, facilitating the evolutionary transition of Heteroptera from herbivorous ancestors to the more complex carnivorous and bloodsucking feeding strategies [2,15,16,17]. Recently, more than 200 proteinaceous components have been deciphered in venom of over 20 heteropterans [6,7,18,19,20,21,22,23,24,25,26]. Few of them have been revealed to participate in multiple biological activities, including extraoral digestion, paralyzing and killing prey, overcoming plant defenses, hemolysis, antibacterial effects, anticoagulation, and anti-inflammation [7,11,20,21,25]. Recent studies indicate that the venom components and functions of the AMG, PMG, and AG differ significantly [5,7]. PMG venom mainly acts through digestive hydrolysis, and this venom have been found to exert neurotoxic, hemolytic, antimicrobial, and cytotoxic activities in some species [5,6,7,20,21,24]. Research confirms that the crude venom extracts of AMG and AG have a unique activity: AMG venom is used to immobilize prey due to its paralyzing function, while the AG venom of most species is inactive, showing insecticidal activity only in *Sycanus bifidus* [7,21,27]. Describing the external morphology and internal ultrastructural characteristics of the three glands (AMG, PMG, and AG) of the venom apparatus will lay a cellular basis for understanding the components and functions that differ in these distinct glands.

The predatory stink bug *Arma custos* (Hemiptera: Pentatomidae: Asopinae) is capable of effectively predating various agricultural and forestry pests, including representatives of Lepidoptera, Coleoptera, and Hymenoptera insects [28,29]. Currently, this species is being mass-produced in factories in China, as well as being used as a biological control agent for controlling several notorious pests. Although the biological activity of *A. custos* crude venom extracts and a few venom proteins have been documented [25,30], the morphological characteristics of its venom apparatus remain undescribed. This study combines light microscopy and electron microscopy techniques to elucidate the morphological and ultrastructural characteristics of the venom apparatus of *A. custos*. Here, we report the structural features of the venom glands and ducts comprising its venom apparatus.

## 2. Materials and Methods

### 2.1. Insects

The *A. custos* colony utilized in this study was sourced from a collection of adult bugs in the suburbs of Kunming, Yunnan Province, China. It has been maintained in our laboratory as detailed by Wang et al. [31]. The bugs were fed a diet with the larvae of the yellow mealworm *Tenebrio molitor*, the greater wax moth *Galleria mellonella*, and the fall armyworm *Spodoptera frugiperda*. Rearing was conducted in cages measuring 40 cm × 40 cm × 40 cm, constructed with nylon netting (44 × 32 mesh) on all sides, accommodating approximately 100 individuals per cage. Soybean plants were included within the cages to provide perching sites. The rearing conditions were maintained at a constant temperature of 25 ± 1 °C, 70 ± 5% relative humidity, and a photoperiod of 14 h light and 10 h dark (14L:10D).

### 2.2. Life Cycle

The life cycle of *A. custos* was observed under a stereo microscope (Leica S6E, Leica, Wetzlar, Germany). When taking photos for the nymphs and adults of *A. custos*, bugs were anesthetized with carbon dioxide and positioned under a stereo microscope using forceps to adjust their posture. But the egg of this bug was photographed directly under a stereo microscope. Imaging of the egg, nymph stages at five different developmental stages, and adult was conducted using a camera system (Leica S6E microscope with digital imaging, Leica, Wetzlar, Germany).

### 2.3. Light Morphology

The gross morphological structure of the venom apparatus of *A. custos* was observed under a stereo microscope (Leica S6E, Leica, Wetzlar, Germany) following the methods described by Su [32]. Briefly, the nymphs and adults of *A. custos* were anesthetized with low-concentration carbon dioxide for 10 min, followed by disinfecting with 75% ethanol. They were dissected in phosphate buffer solution (PBS, pH 7.4) under a stereo microscope. During dissection, use forceps to tear along the ventral margin of the body, removing other tissues to fully expose the venom apparatus. Then, the whole venom apparatus was imaged using a camera system (Leica S6E microscope with digital imaging).

### 2.4. Electron Microscopy

Following the method described in Section 2.3, various venom glands, including AMG, PMG and AG, as well as associated ducts including the venom duct connecting the main gland (VD), the anterior of the duct connecting the accessory gland to the main gland (AAMD), and the posterior of the duct connecting the accessory gland to the main gland (PAMD), were collected into 1.5 mL microcentrifuge tubes, respectively. For cross-section analysis, these glands and ducts underwent electron microscopy analysis following the procedures detailed by Wu [7]. The above six kinds of sample tissues were randomly selected from three samples of each of the five insects for observation, and observations and photography were carried out from the cells to the cavity from all around the cross-section of the sample.

### 2.5. Image Visualization

Images were visualized and processed with Adobe Photoshop 7.0 (Adobe Systems Inc., San Jose, CA, USA) and Adobe Illustrator CS6 (Adobe Systems Inc., San Jose, CA, USA).

## 3. Results

### 3.1. Gross Morphology of the Venom Apparatus

**Figure 1:**
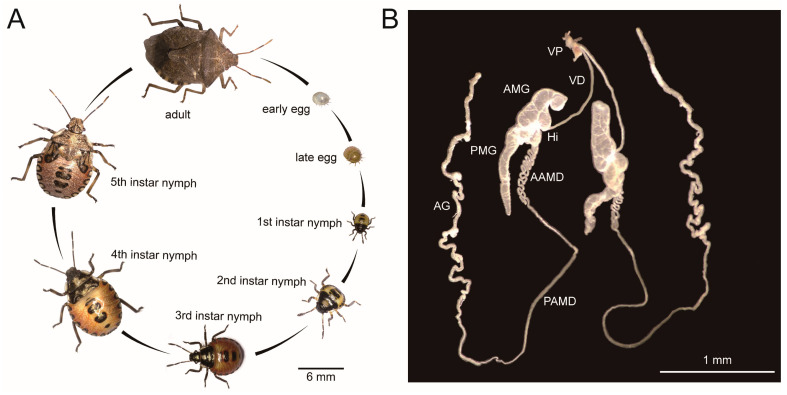
Life cycle (**A**) and gross morphology of the venom apparatus (**B**) of *Arma custos*. VP: venom pump; VD: venom duct connecting the main gland; AMG: anterior main gland; Hi: hilum; PMG: posterior main gland; AAMD: anterior of the duct connecting the accessory gland to the main gland; PAMD: posterior of the duct connecting the accessory gland to the main gland; AG: accessory gland.

The venom apparatus of *A. custos* is primarily situated in the thorax, maintaining a consistent morphology from nymph to adult while progressively increasing in size with developmental stages (Figure 1A and Figure S1). The *A. custos* venom apparatus consists of a pair of main gland and accessory gland (Figure 1B). The main gland consist of two lobes, with the anterior main gland (AMG) shorter than the posterior main gland (PMG). Between the two lobes of the main gland, there is a strong constriction, characterizing a hilum (Hi) where we see the insertion of two narrow and independent ducts, the venom duct connecting the main gland (VD) and the duct connecting the accessory gland to the main gland (AMD), respectively. The venom duct (VD) extends from the hilum toward the head and connects with the venom pump (VP) and the mouth needle. The AMD is divided into two sections due to morphological differences: the anterior of the duct connecting the accessory gland to the main gland (AAMD) is U-shaped and folded, and the posterior of the duct connecting the accessory gland to the main gland (PAMD) is straight and morphologically similar to the VD. The tubular accessory gland (AGs) formed by PAMD extend toward the thoracic cavity and are irregularly curved (Figure 1B). Both AMG and PMG are characterized by a prominent sac-like shape, providing a significant reservoir for venom storage, compared to the tubular AG.

### 3.2. Ultrastructure of AMG

**Figure 2:**
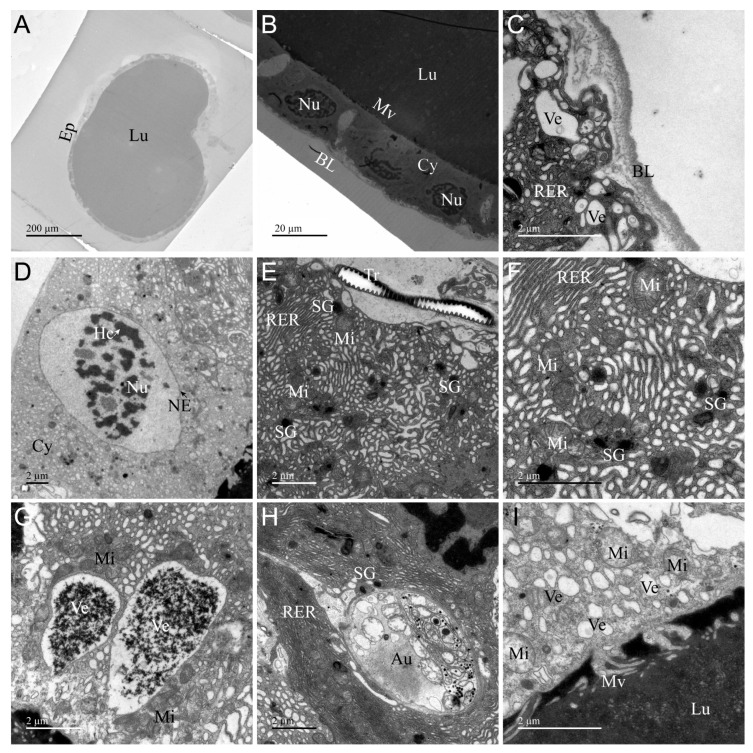
Transmission electron micrographs of the anterior main gland (AMG) of *Arma custos*. (**A**) Comprehensive view of the ultrastructure; (**B**) Localized view of epithelial cells; (**C**) Basal cell region; (**D**–**H**) Median cell region; (**I**) Apical cell region. Au: autophagosome; BL: basal lamina; Cy: cytoplasm; Ep: epithelial; Hc: heterochromatin (white arrow); Lu: lumen; Mi: mitochondrion; Mv: microvillus; NE: nuclear envelope (black arrow); Nu: nucleus; RER: rough endoplasmic reticulum; SG: secretory granule; Tr: trachea; Ve: vesicle.

The ultrastructure of AMG of *A. custos* is shown in Figure 2. It consists of a layer of cuboidal secretory cells encircling a large lumen densely filled with electron-dense material, indicative of venom storage (Figure 2A). The basal regions of these secretory cells are enveloped by a thick, extensible basal placode structure, yet the plasma membrane does not exhibit infoldings (Figure 2B,C). A well-developed rough endoplasmic reticulum is present in the cytoplasm, alongside nuclei, secretion granules, mitochondria, secretory vesicles, tracheae, and autophagosomes (Figure 2D–H). The apical surface of the secretory cells is adorned with microvilli (Figure 2B,H). Centrally located within the secretory cell is a spherical or oval nucleus characterized by a prominent nuclear envelope and dispersed chromatin (Figure 2D). In the secretory cells, compared to the basal and apical secretory vesicles, those in the intermediate region are larger in volume and their contents fill the entire vesicle lumen (Figure 2C,G,I). Furthermore, the electron density distribution within secretory vesicles is uneven, resembling a granular pattern (Figure 2G). Secretory granules are scattered throughout the cytoplasm and are present in small numbers (Figure 2E,F,H). Autophagosomes in the cytoplasm encapsulate organelles, proteins, and other components requiring degradation (Figure 2H). AMG exhibits the typical characteristics of secretory cells, performing the function of synthesizing and secreting venom, while its extensive lumen serves as a storage site for the venom.

### 3.3. Ultrastructure of PMG

**Figure 3:**
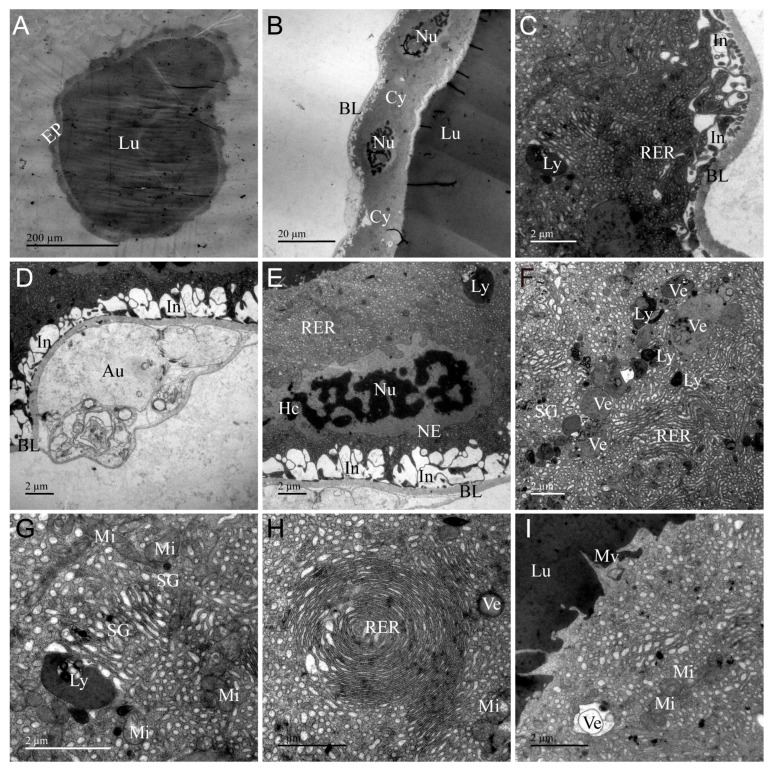
Transmission electron micrographs of the posterior main gland (PMG) of *Arma custos*. (**A**) Comprehensive view of the ultrastructure; (**B**) Localized view of epithelial cell; (**C**) Basal cell region; (**D**–**H**) Median cell region; (**I**) Apical cell region. Au: autophagosome; BL: basal lamina; Cy: cytoplasm; Ep: epithelial; Hc: heterochromatin; In: infolding; Lu: lumen; Ly: lysosomes; Mi: mitochondrion; Mv: microvillus; NE: nuclear envelope; Nu: nucleus; RER: rough endoplasmic reticulum; SG: secretory granule; Ve: vesicle.

The ultrastructure of PMG of *A. custos* is shown in Figure 3. It consists of a layer of cuboidal secretory cells encircling a large lumen densely filled with electron-dense material, which stores a significant amount of venom (Figure 3A). The boundaries between these secretory cells are indistinct (Figure 3B). The basal region of these secretory cells is encased by a layer of extensible basal placodes, and the plasma membrane exhibits numerous infoldings (Figure 3C–E). The PMG basal placode is relatively thick and displays a bilayer structure in certain areas (Figure 3D). In the median region, a well-developed rough endoplasmic reticulum is present in the cytoplasm, alongside mitochondria, nuclei, secretory vesicles, autophagosomes, lysosomes, tracheae, and secretion granules (Figure 3E–H). The apical surface of the secretory cells is adorned with microvilli of various shapes (Figure 3I). The nuclei of secretory cells in the PMG are rectangular in shape, characterized by irregularly shaped nuclear membranes and condensed chromatin (Figure 3E). Interestingly, secretory vesicles are arranged in an orderly fashion within specific regions of the cytoplasm, with their lumens filled with a substance of uniform texture (Figure 3F). Additionally, a ring-shaped rough endoplasmic reticulum in certain regions was observed to be densely packed throughout the cytoplasm, indicating that this area possesses a high capacity for synthesizing and secreting proteins (Figure 3H). The ultrastructural characteristics of the PMG indicate that this gland is the site where venom is secreted, synthesized, and stored.

### 3.4. Ultrastructure of AG

**Figure 4:**
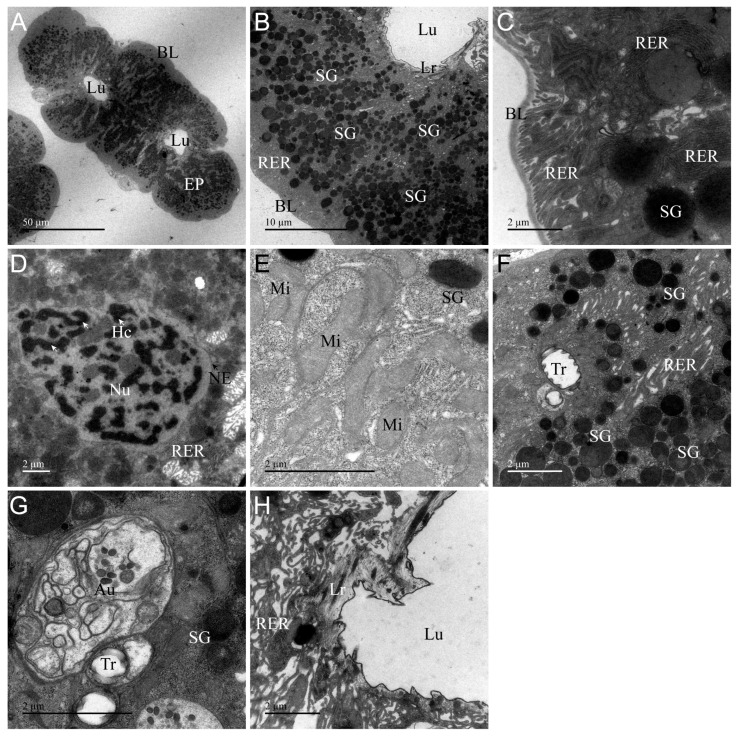
Transmission electron micrographs of the accessory gland (AG) of *Arma custos*. (**A**) Comprehensive view of the ultrastructure; (**B**) Localized view of epithelial cell; (**C**) Basal cell region; (**D**–**G**) Median cell region; (**H**) Apical cell region. Au: autophagosome; BL: basal lamina; Ep: epithelial; Hc: heterochromatin (white arrow); Lr: layer; Lu: lumen; Mi: mitochondrion; NE: nuclear envelope (black arrow); Nu: nucleus; RER: rough endoplasmic reticulum; SG: secretory granule; Tr: trachea.

The ultrastructural features of AG of *A. custos* are shown in Figure 4. This gland is composed of irregularly shaped secretory cells that form two narrow and small lumens (Figure 4A). The basal placodes in the basal region of the AG are thin and tightly bound to the surface of secretory cells (Figure 4C). The organelles are similar to those found in the main gland, but the morphology of some organelles in the AG exhibits significant differences. In AG, secretory granules are arranged more densely and are larger in volume. Chromatin is dispersed throughout the entire nucleus; mitochondria appear swollen. Autophagosomes are smaller in volume (Figure 4B,D,E,G). Additionally, AG lacks microvilli and secretory vesicles and features a thick heterogeneous layer with uneven electron density at the apical region of the cells (Figure 4A,F,H). This indicates that AG possesses the capacity to synthesize and secrete venom, while its narrow sac cavity limits venom storage.

### 3.5. Ultrastructure of VD

**Figure 5:**
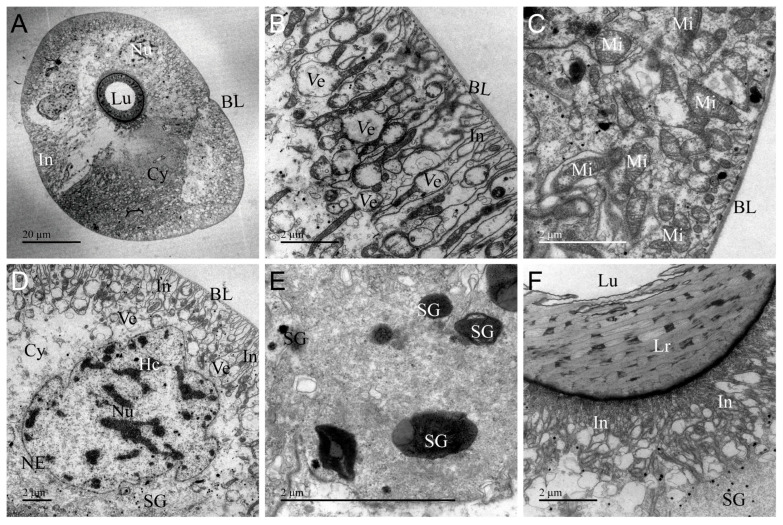
Transmission electron micrographs of the venom duct of main gland (VD) of *Arma custos*. (**A**) Comprehensive view of the ultrastructure; (**B**) Basal cell region; (**C**–**E**) Median cell region; (**F**) Apical cell region. BL: basal lamina; Cy: cytoplasm; Hc: heterochromatin; In: infolding; Lr: layer; Lu: lumen; Mi: mitochondrion; NE: nuclear envelope; Nu: nucleus; SG: secretory granule; Ve: vesicle.

In the venom apparatus, VD is the duct connecting the main gland to the venom pump. The ultrastructure of VD reveals epithelial cells arranged in elliptical shapes, forming a narrow and rounded lumen (Figure 5A). The plasma membrane of the epithelial cells displays extensive infoldings at both the basal and apical regions, creating enlarged extracellular channels that enhance intercellular communication and transport (Figure 5B,F). Only the secretory vesicles, mitochondria, nucleus, and a few secretory granules are found in the cytoplasm (Figure 5B–E). Mitochondria and plasma membranes are alternately distributed in the basal region (Figure 5C). The cell nucleus is irregular in shape, with heterochromatin abnormally dispersed within it (Figure 5D). Beyond the apical region of epithelial cells lies a heterogeneous layer of varying electron density that separates the delivered venom from the epithelial cells, thereby protecting the host tissue from toxicity (Figure 5F). The VD is primarily responsible for venom transport, featuring well-developed plasma membrane infoldings and mitochondrial structures that provide the necessary energy for supporting venom delivery.

### 3.6. Ultrastructure of AMD

**Figure 6:**
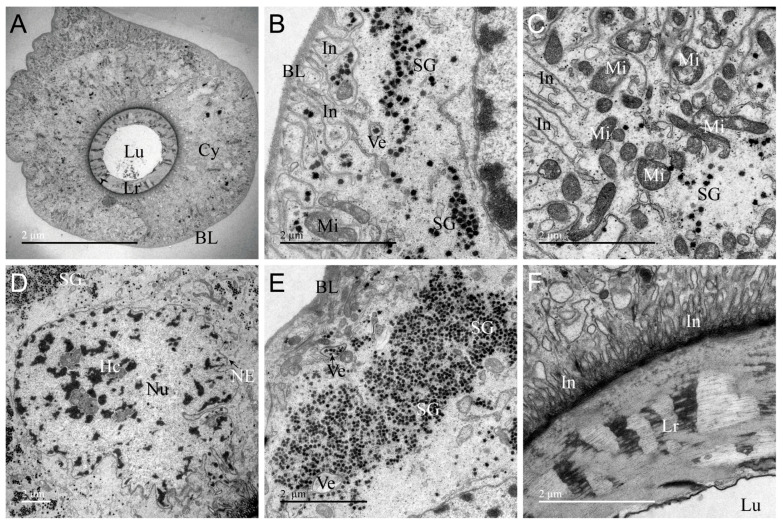
Transmission electron micrographs of the anterior of the duct connecting the accessory gland to the main gland (AAMD) of *Arma custos*. (**A**) Comprehensive view of the ultrastructure; (**B**) Basal cell region; (**C**–**E**) Median cell region; (**F**) Apical cell region. BL: basal lamina; Cy: cytoplasm; Hc: heterochromatin; In: infolding; Lr: layer; Lu: lumen; Mi: mitochondrion; NE: nuclear envelope; Nu: nucleus; SG: secretory granule; Ve: vesicle.

AMD of the duct connecting the AG to the main gland is classified as AAMD or PAMD based on morphological differences. The ultrastructure of AAMD observed by transmission electron microscopy is shown in Figure 6. Longitudinal sections reveal epithelial cells arranged in an elliptical pattern, forming a narrow circular lumen (Figure 6A). The plasma membrane of epithelial secretory cells displays extensive infoldings at both the basal and apical regions (Figure 6B,F). In the basal region, these plasma membranes extend longer and are long and sparsely distributed, forming larger channels that facilitate the movement of substances and the exchange of information between the intracellular and external environment (Figure 6B). In contrast, the plasma membrane in the apical region exhibits a shorter and more densely packed arrangement (Figure 6F). In the central region, in addition to mitochondria, nuclei, and secretory vesicles, numerous small and densely distributed secretory granules are observed, migrating gradually toward the basal region along the plasma membrane (Figure 6C–E).

**Figure 7:**
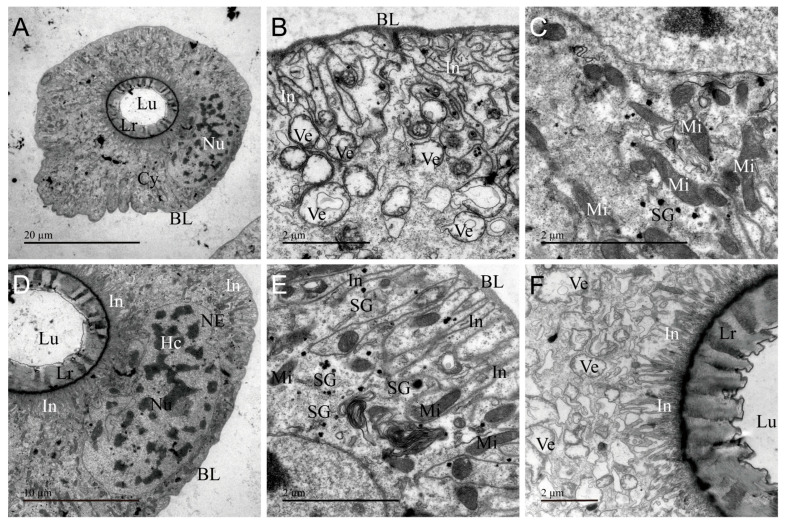
Transmission electron micrographs of the posterior of the duct connecting the accessory gland to the main gland (PAMD) of *Arma custos*. (**A**) Comprehensive view of the ultrastructure; (**B**) Basal cell region; (**C**–**E**) Median cell region; (**F**) Apical cell region. BL: basal lamina; Cy: cytoplasm; Hc: heterochromatin; In: infolding; Lr: layer; Lu: lumen; Mi: mitochondrion; NE: nuclear envelope; Nu: nucleus; SG: secretory granule; Ve: vesicle.

The ultrastructural features of PAMD are illustrated in Figure 7. Epithelial cells of PAMD exhibit spherical elliptical shapes, forming a narrow lumen (Figure 7A). Similar to VD and AAMD, the plasma membranes of epithelial cells in PAMD exhibit extensive infoldings at both the basal and apical regions (Figure 7B,F). The cytoplasm contains only secretory vesicles, secretion granules, nuclei, and a substantial number of mitochondria but lacks typical secretory organelles such as the rough endoplasmic reticulum (Figure 7B–E). The cell nucleus is well-developed and nearly square in shape, with heterochromatin distributed throughout the entire nucleus (Figure 7D). In AG, VD, and AMD, the apical region of the cells is lined externally with layers of different electron densities, where the edges and inter-regions of the layers are locally dense in electron density, while the other intermediate areas have lighter electron density (Figure 4H, Figure 5F, Figure 6F and Figure 7F). AMD primarily facilitates venom transport, with the well-developed plasma membrane infoldings and mitochondria in epithelial cells serving as structural features for material transport.

### 3.7. Differences and Similarities in the Ultrastructure of Various Organs

To sum up, we systematically analyzed the ultrastructural characteristics between glands, ducts, as well as glands and ducts. Compared with the glands, the ultrastructure of AMG and PMG was basically the same, and there was a significant difference from AG. The difference between AMG and PMG was in the nucleus, vesicles, and microvilli (Figure 2 and Figure 3). The nucleus in the AMG was oval and the chromatin was scattered, while in the PMG, it was rectangular and the chromatin was aggregated (Figure 2D and Figure 3E); the vesicles in the AMG were fewer in number and larger in size, with an uneven electron density, while the PMG showed the opposite (Figure 2G and Figure 3F); the microvilli in the AMG were clustered, and in the PMG, they were single and apically sharp (Figure 2I and Figure 3I). Compared with the main gland, the accessory gland has two narrow lumens (Figure 2A, Figure 3A and Figure 4A), the secretory cells lack secretory vesicles and microvilli, a large number of secretory granules are distributed in the cytoplasm (Figure 4B,F), and the apical region is lined with a layer of uniform dense electrons (Figure 4H). Although all three glands are capable of secreting venom, the venom is primarily stored in the lumens of the AMG and PMG.

The ducts’ (VD and AMD) ultrastructure characteristics are similar: the basal and apical regions of the infoldings are well developed, and only the nucleus, mitochondria, vesicles, and secretory granules are present in the cytoplasm (Figure 5, Figure 6 and Figure 7). This is a cell structure feature responsible for the transport of substances. Interestingly, the distribution of secretory granules is inconsistent in VD, AAMD, and PAMD, with sporadic distribution in VD and PAMD and dense distribution in local areas of AAMD (Figure 5E,F, Figure 6B,C,E and Figure 7C,E). Furthermore, the apical region of the cell is bordered by an electron-dense layer (Figure 5A,F, Figure 6A,F and Figure 7A,F).

The types of organelles in the glandular and tubular cells were obviously different; for example, we found that organelles such as lysosomes, autophagosomes, rough endoplasmic reticulum, and microvilli were only present in the glandular cells, while the other organs and infoldings were present in both the glandular and tubular cells (Figure 2, Figure 3, Figure 4, Figure 5, Figure 6 and Figure 7). Meanwhile, some of the coexisting organelles had different morphological characteristics, as the vesicles were of higher electron density in the main gland than in the AG and ducts, and were filled with venom proteins; the secretory granules were larger in volume than the glandular cells. The presence or absence of infolding is a major structural feature of the main glands, the accessory glands, and the duct cells, which are present in the basal and apical regions of the duct cells, in the basal region only of the PMG, and in none of the AMG and accessory glands (Figure 2C, Figure 3C, Figure 4C, Figure 5B,F and Figure 6B,F). Furthermore, layers of varying electron density cover the outer apical regions of the accessory glands and duct cells. Notably, this layer appears as an irregular ring in accessory glands, whereas in the ducts, it forms a circular ring (Figure 4A, Figure 5A, Figure 6A and Figure 7A), and near the lumen edge, the layer shows teeth of varying sizes (Figure 4H, Figure 5F, Figure 6F and Figure 7F). These results indicate that venom proteins are synthesized and stored by the AMG, PMG, and AG, while the VD and AMD ducts are responsible for transporting the venom.

## 4. Discussion

The venom apparatus of *A. custos* comprises paired bilobed main glands (AMG and PMG) and an elongated tubular AG, anatomically resembling those found in other stink bugs [11,13,33,34]. The venom glands in heteropterans vary in shape and number of lobes, especially in the number of MG lobes and the morphology of AGs [8,12,14,35]. So far, the main gland are mostly bilobed, but there are also single-lobed, trilobed, and multilobed forms [10,14,35]. AMG and PMG have similar morphological structures in all species of main gland bilobed heteropterans, with the AMG being short and heart-shaped and the PMG extending toward the abdomen in a long sac-like form [7,8,34]. Interestingly, AG shows great morphological diversity, such as tubular and vesicular forms [7,10,34]. In addition, the tubular AGs also exhibit subtle differences in narrowness and width [8,34].

Ultrastructural examination of the venom glands in *A. custos* reveals that the AMG and PMG consist of a layer of cubic or spherical glandular cells forming a large circular lumen, while the AG exhibits two narrow lumens. The glandular cells of the AMG, PMG, and AG are characterized by rich organelles such as nuclei, endoplasmic reticulum, mitochondria, and secretory vesicles, typical features of secretory cells. All three glands are capable of venom secretion, and their lumens are primarily used for venom storage [12,34]. In glandular cells, the thickness of the basal lamina varies across different glands and ducts. The basal lamina of AMG and PMG is thicker than that of AG, VD, and AMD. Additionally, the PMG basal lamina exhibits distinct bilayer structural characteristics, whereas the basal lamina in the AG and venom gland ducts is thinner and closely adheres to the cell surface. It has been confirmed that the basal lamina originates from two distinct polymer networks: one composed of laminin and the other of type IV collagen [36]. Laminin is tightly associated with the cell surface, while the type IV collagen network in basal lamina is covalently linked together by multiple chemical bonds that are thought to confer the tensile strength of the basal lamina [36]. Therefore, we hypothesize that the composition of the basal lamina in AMG and PMG differs from that in AG and ducts. The ultrastructure can only reveal differences in thickness but cannot definitively determine its composition. Additionally, some studies have confirmed the mechanical properties of the basal lamina, such as tensile strength and stiffness, through mechanical testing [37,38]. According to the ultrastructural characteristics of AMG and PMG, they serve as the primary venom storage sites, with their thicker basement membranes capable of withstanding the pressure exerted by venom storage that causes cellular deformation.

The ultrastructural analysis demonstrates heterogeneity in organelle morphology and composition within the secretory cells of the AMG, PMG, and AG. Specifically, the glandular cells of the PMG exhibit columnar nuclei with condensed chromatin, while those of the AMG and AG possess elliptical nuclei with dispersed chromatin in a heterochromatic state. Like our predecessors, we also hypothesize that condensed chromatin is associated with cells exhibiting high metabolic activity involved in protein synthesis and RNA production [9,13,39,40]. Furthermore, ultrastructural analysis reveals that AMG and PMG primarily transport secretory proteins via secretory vesicles, whereas AG mainly utilizes secretory granules for transport. Interestingly, the secreted proteins within the vesicles or granules of AMG, PMG, and AG exhibited distinct characteristics. AMG vesicles showed non-uniform electron density with a granular appearance, while PMG vesicles and AG secretory granules displayed uniform electron density but with varying degrees of color intensity. Additionally, the varying electron densities in the ultrastructure of the AMG, PMG, and AG indicate the synthesis of different compounds [8,12,41]. To date, only two studies have reported on the components and functions of *A. custos* crude venom, but the components and functions of AMG, PMG, and AG venoms remain unclear [25,30]. At the same time, some studies have also shown that there are significant differences in the venom composition and function of these three glands: PMG venom is used for digestion and hydrolysis, AMG venom is used to paralyze prey, and AG venom is inactive in most species [5,6,7,21,24]. PMG venom may contain components related to digestive hydrolysis, such as serine proteases, cathepsins and various other digestive hydrolases, but further investigations are needed to determine which specific enzymes perform this function [7,21]. In contrast, AMG venom can paralyze prey, and its components are likely primarily neurotoxins and similar substances [7,21,27]. These findings highlight the ultrastructural distinctions among AMG, PMG, and AG of *A. custos*, which likely contribute to variations in the synthesis of venom protein components and biological activity across them. In addition, the apical regions of AG, VD, and AMD cells are lined by a layer of uneven electron density, with the layer likely serving to isolate venom within the lumen during transport, thereby protecting secretory cells and preventing self-poisoning [39,41].

Transmission electron microscopy examination of the duct of venom glands in *A. custos* reveals that the VD, AAMD, and PAMD consist of a layer of cubic or spherical glandular cells forming a ring-shaped channel. The microstructural features of these ducts closely resemble those of AD, except for the absence of endoplasmic reticulum and autophagosomes, but exhibit well-developed plasma membrane infoldings and mitochondria. The highly developed plasma membrane infoldings and mitochondria facilitate material exchange with the hemolymph, providing the energy support necessary for venom transport [14,42,43,44]. This is similar to previous findings that ducts of the venom apparatus of the bugs lack secretory function and are primarily responsible for the rapid and precise delivery of venom [42,43]. The duct is mainly used to deliver venom, but it can avoid self-poisoning, which may be related to the special structure within the duct. Electron microscopy of the entire duct shows that a layer of uneven electron density exists at the apical surface of the duct epithelial cells, functioning as a natural barrier that separates the transported venom from the cells, thereby preventing self-poisoning.

## 5. Conclusions

This study provides a detailed examination of the morphology and ultrastructure of the venom apparatus of *A. custos*. It is a complex venom apparatus, with each venom gland and duct tissue having distinct functions and clear divisions of labor. Among them, AMG, PMG, and AG are the sites for venom proteins synthesis and storage, while VD and AMD are responsible for venom delivery. In addition, the ultrastructural differences between AMG, PMG, and AG indicate that each of them can produce distinct secretions leading to the compositional and functional heterogeneity associated with these venom glands.

## Data Availability

The original contributions presented in this study are included in the article/Supplementary Material. Further inquiries can be directed to the corresponding author.

## Supplementary Materials

The following supporting information can be downloaded at https://www.mdpi.com/article/10.3390/insects17030340/s1, Figure S1. Morphology of the venom apparatus of *Arma custos* across developmental stages. N1: 1st instar nymph; N2: 2nd instar nymph; N3: 3rd instar nymph; N4: 4th instar nymph; N5: 5th instar nymph; A: adult.
