# Supplementary figures and images for: Morphological and Ultrastructural Characterization of the Venom Apparatus of the Predatory Stink Bug, Arma custos
Source: Insects. 2026 Mar 20;17(3):340. doi: 10.3390/insects17030340 (PMC13026130; doi:10.3390/insects17030340)

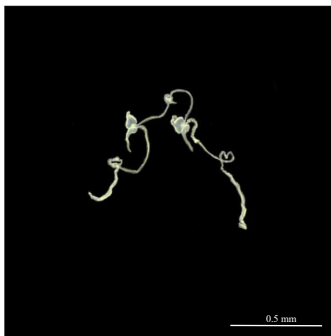

N1

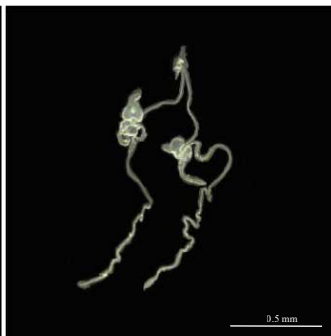

N2

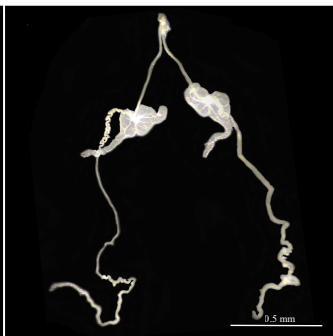

N3

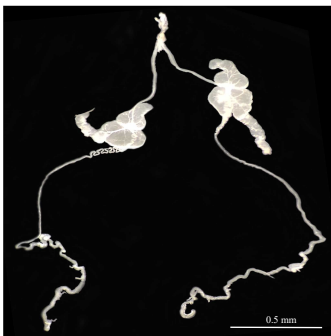

N4

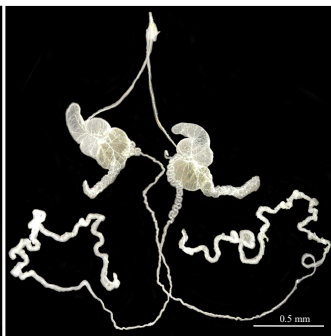

N5

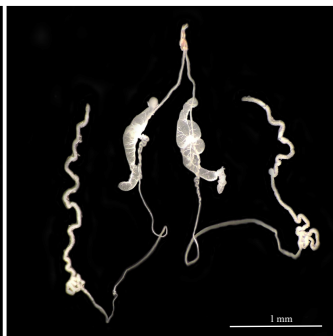

A

Supplement: Supplementary file 1 [file insects-17-00340-s001.zip › insects-4147441-supplementary.pdf]
